# Withholding Stress Ulcer Prophylaxis To Mechanically Ventilated Enterally-Fed Critically Ill Patients Appears Safe: A Randomised Double-Blind Placebo Controlled Pilot Study

**DOI:** 10.1186/2197-425X-3-S1-A41

**Published:** 2015-10-01

**Authors:** SP Selvanderan, MJ Summers, MP Plummer, ME Finnis, Y Ali Abdelhamid, MB Anderson, MJ Chapman, CK Rayner, AM Deane

**Affiliations:** The University of Adelaide, Discipline of Acute Care Medicine, Adelaide, Australia; Royal Adelaide Hospital, Intensive Care Unit, Adelaide, Australia; The University of Adelaide, Discipline of Medicine, Adelaide, Australia; Royal Adelaide Hospital, Department of Gastroenterology and Hepatology, Adelaide, Australia

## Introduction

Acid-suppressing drugs are routinely prescribed to mechanically ventilated patients for prophylaxis against stress ulceration, but there is a lack of recent data to support this strategy.

## Objectives

To evaluate the effect of routine pantoprazole administration on rates of clinically significant gastrointestinal (GI) bleeding and *Clostridium difficile* infection in mechanically ventilated patients suitable for enteral nutrition.

## Methods

This prospective randomised double-blind study was performed over a 12-month period. All critically ill patients who were anticipated to be ventilated for greater than 24 hours and enterally fed within 48 hours were eligible. Patients receiving acid-suppressing medication prior to admission and those admitted with GI bleeding were excluded. Patients were assigned to receive IV pantoprazole or placebo daily until no longer mechanically ventilated, or for a maximum of 14 days. Data were collected for overt GI bleeding (haematemesis, blood-stained gastric aspirate, melaena or haematochezia), clinically significant bleeding (overt bleeding accompanied by a reduction in mean arterial pressure >20mmHg in the absence of another cause, or reduction in haemoglobin >20g/L, or need for endoscopy/surgical intervention) and *C. difficile* infection. Patients were tested for *C. difficile* if they had diarrhoea (defined as ≥3 bowel movements in 24 hours) or a temperature of ≥38.6°C and white cell count ≥20 x 10^9^/mL. Data are mean ± SD or median (IQR) and compared using chi-squared tests or Mann-Whitney U tests as appropriate.

## Results

Two hundred and nine patients were assigned to either pantoprazole (n = 104) or placebo (n = 105) in a randomised double-blind fashion, with 1623 ventilation days and 2301 ICU treatment days observed. The pantoprazole and placebo groups were similar in terms of age (52 ± 18 vs. 51 ± 18 years) and APACHE II scores (19.6 ± 6.4 vs. 18.8 ± 7.2). There were no episodes of clinically significant bleeding in either group. Three patients (2.9%) assigned pantoprazole and five patients (4.7%) receiving placebo had an episode of overt bleeding (p = 0.48). Thirty patients assigned to pantoprazole and forty assigned to placebo were tested for *C. difficile* with only one patient (receiving pantoprazole) diagnosed with *C. difficile* infection.

## Conclusions

Clinically significant GI bleeding appears to occur rarely in mechanically ventilated patients who are expected to receive enteral nutrition. Accordingly, the capacity for such patients to benefit from prophylactic administration of an acid-suppressive drug appears limited. Further study to evaluate the generalisability of these data is required.

## Grant Acknowledgment

This project was funded by a Royal Adelaide Hospital Research Foundation project grant.

